# The Body in Neurosciences: Representation, Perception and Space Processing

**DOI:** 10.3390/brainsci13121708

**Published:** 2023-12-12

**Authors:** Liana Palermo, Maddalena Boccia

**Affiliations:** 1 Department of Medical and Surgical Sciences, Magna Graecia University of Catanzaro, Viale Europa—Loc. Germaneto, 88100 Catanzaro, Italy; 2 Department of Psychology, “Sapienza” University of Rome, via dei Marsi 78, 00185 Rome, Italy; maddalena.boccia@uniroma1.it; 3 Cognitive and Motor Rehabilitation and Neuroimaging Unit, IRCCS Santa Lucia Foundation, via Ardeatina 306, 00179 Rome, Italy

The Special Issue “The Body in Neurosciences: Representation, Perception and Space Processing” deals with the understanding of body processing in terms of the multisensorial perception of bodily related information, interoception, and mental representation, as well as its relationship with the peripersonal, interpersonal, and extrapersonal spaces, integrating findings from normal and pathological functioning.

The singular nature of the body, which is an object that can be perceived from both the inside and the outside, has long captivated the neuroscience community. Several empirical findings ([1,2,3,4,5]; for an overview, see also [6,7,8]) converged on the idea that exteroceptive and interoceptive bodily signals are fundamental for the development of body representations and self-consciousness (in this Special Issue, see, for example, Raimo et al. [9] for findings on interoceptive bodily signals, or Spitoni et al. [10] for findings on touch and vision). The perception of different bodily signals and their integrated neural representation also play a role in mental health [11] and other aspects of cognition (in this Special Issue, see, for example, the studies by Fanghella et al. [12] and Hatzipanayioti and Avraamides [13]), ranging from processing the peripersonal [14,15] and navigational spaces [16] to processing interpersonal body representations [17,18] and understanding others’ emotional experiences [19,20].

Still, a convergent view of body processing and its development, which integrates the perceptual and representational levels and interaction with the surrounding space, is missing. For this reason, we launched this Special Issue, calling for new contributions on the topic. This Special Issue includes eight studies that, using systematic review, behavioral, neuroimaging, and neuropsychological approaches, provide new insights into the development, neural bases, cognitive mechanisms, and disorders of body processing and the relation with different kinds of space.

In the last decade, several studies have identified the sense of touch as a valuable modality for investigating body representations [10,21,22,23]. Along these lines, Gambino et al. [24] offered a novel contribution to disclose the interaction between body representations and haptic perception, studying extreme obesity. Following Tamé et al. [25], different forms of perception involve referencing sensory signals to a stored representation of the body itself. This is particularly true for touch, since the skin (i.e., the primary receptor surface) is “physically co-extensive with the body itself”. In this vein, Gambino et al. [24] hypothesized that individuals with altered body representation, such as severe obesity, could show difficulties in haptic perception. Both behavioral and electrophysiological data confirmed this prediction. Specifically, the quality of haptic performance in the group of individuals with severe obesity was poorer than in control participants. Also, a significant decrease in theta, alpha, and beta frequencies in the right temporo-parietal areas and a significant increase in the gamma bands in the left frontal areas were found in the group of individuals with severe obesity. These findings suggest that severe obesity could result in reduced haptic performances and atypical activation of brain areas underlying multisensory integration.

Spitoni et al. [10] focused on a specific dimension of body representation, namely the “Metric Component of the Body Representation” (MCBR). The MCBR is used to process the discrimination of tactile distance, and, at the neural level, previous evidence suggests that the right angular gyrus subserves it. The authors replicated this finding with a new tDCS study and, in addition, aimed to understand whether the right angular gyrus is also implicated in the visual metric component of body representation. They presented a tDCS study on healthy volunteers, in which the right angular gyrus was perturbed during a visual distance discrimination task in which visual stimuli were delivered by a laser projector on the body or the desk. In both conditions, the participant’s task was to evaluate which of the two distances was bigger. In contrast to the sham stimulation, the anodal stimulation of the right angular gyrus led to faster vocal response times, indicating that this brain area also discriminates visual distances on the body.

Spaccasassi et al. [26] examined the relationship between affective touch, on the one hand, and body representation and the space around our body (peripersonal space) on the other. Affective touch, with gentle and slow skin stroking accompanied by pleasant sensations, is considered a skin-mediated interoceptive modality [27,28]. Concerning body representation, the study focused on a specific aspect, namely, body ownership—the experience of the body as one’s own—using a well-known experimental paradigm, the rubber hand illusion (RHI). There is mixed evidence in the literature about the role of affective touch in enhancing the embodiment of a fake hand compared to neutral touch using the RHI. The behavioral and physiological data by Spaccasassi et al. [26] suggest that affective touch does not modulate body ownership. Similarly, they did not find a role of the affective touch in modulating the peripersonal space. The interindividual variability in this specific interoceptive modality could partly account for differences across studies; indeed, individual differences in interoception play a role in different cognitive and affective processes, as reported, for example, by Raimo et al. [9].

Specifically, Raimo et al. [9] provided new insight into the development of functional body representations across the lifespan and their relationship with the interindividual variability in a specific interoceptive dimension, interoceptive sensibility (ISe). ISe is defined as the dispositional tendency to focus on interoceptive signals and is typically assessed using self-report questionnaires [29,30]. Concerning functional body representations, they investigated action-oriented (i.e., body schema) and nonaction-oriented body representations. The findings from a sample of 239 healthy participants divided into five age groups (7–8 yrs.; 9–10 yrs.; 18–40 yrs; 41–60 yrs.; over 60 yrs.) suggest that action- and nonaction-oriented body representations follow an inverted U-shaped developmental curve, with younger children (7–8 yrs.) and older adults (over 60 yrs.) performing worse. Body representations, particularly action-oriented body representation, are also negatively affected by higher ISe levels in childhood and late adulthood. This last finding suggests the idea that, in specific periods of life, an excessive focus on interoceptive sensations can have a detrimental effect on body representations.

Di Vita et al. [31] also investigated functional body representations using a developmental neuropsychology perspective. The authors compared the performance of children with cerebral palsy with that of typically developing children, assessing action-oriented (i.e., body schema) and nonaction-oriented body representations (i.e., body semantics and body structural representation), while considering the performance in control tasks involving non-body stimuli. Body representation deficits, mainly involving the body structural representation and body schema, can be frequently detected in cerebral palsy. Also, these impairments could be more evident after eight years of age, when typically developing children start to reach an adult-like pattern of body representation development.

These impairments can significantly impact an individual’s functional independence due to the influence of body representations on the execution of actions. In this vein, these findings may have implications for developing innovative training protocols grounded in a clear understanding of body representation development and deficits in children with cerebral palsy.

A perspective review with intriguing theoretical and clinical implications (e.g., for conditions such as autism spectrum disorder) was presented by Fanghella et al. [12]. In their review, the authors proposed that interpersonal interactions may affect body and peripersonal space representations. The authors first explored how actions modulate the multisensory representations of the body and the peripersonal space, describing how they can be extended to incorporate tools or additional body parts, as during bodily illusions. Then, they introduced the hypothesis of shared body and peripersonal space representations emerging during interpersonal interaction. In particular, they discussed current evidence pointing to the building of joint body and peripersonal space representations when two individuals engage in a task that requires interpersonal sensorimotor coordination. They also discussed how these mechanisms might operate differently in individuals with autism spectrum disorder and how this proposal aligns with recent evidence of peripersonal space rigidity in this disorder and the possible association with difficulties during interpersonal coordination.

In healthy individuals, there is evidence that reaction times for sensory events occurring within the space immediately surrounding the body (i.e., peripersonal space) are faster when compared to the processing of the same sensory events occurring in the extrapersonal space [32]. This can be attributed to the importance of sensory events that take place in the immediate vicinity of the body. Indeed, such events play a critical role in defining the spatial boundaries of the bodily self, facilitating goal-directed actions and detecting potential threats [33]. Instead, little is known about another relevant feature of sensorial stimulation: the size of the stimulated area. Martolini et al. [34] examined whether increasing the size of the stimulated body area enhances or impairs sensory discrimination during unimodal (visual, auditory, tactile) and multimodal stimulation. The authors asked healthy adults to discriminate unimodal and multimodal stimuli produced by one, two, three, or four devices positioned on the forearm. In the unisensory conditions, increasing the stimulated area had a detrimental effect on all kinds of perceptual stimulations (i.e., visual, auditory, and tactile). During multisensory stimulation, integrating auditory and tactile information improved sensory accuracy more than unisensory (audio, tactile) processing but only when the stimulated area increased (i.e., when the stimulation area is augmented to four devices). The authors also suggested opportunities for future research studies that could expand on these findings, considering different developmental stages across the lifespan and the various kinds of space.

Finally, Hatzipanayioti and Avraamides [13] investigated whether blocking visual access to one’s body using virtual reality reduces difficulties in tasks that require adopting a spatial perspective other than the one we occupy physically (i.e., mental perspective taking). Indeed, difficulties could arise when responding from an imagined viewpoint because of the need to inhibit egocentric codes that specify where objects are relative to the observer’s actual position. Participants were asked to imagine themselves occupying various positions around a round table and to point to the position of a virtual character also sitting around the table. The task was performed in a virtual reality condition, where participants could not see their own bodies and arms, and in a real-world condition, where they could. Different from the authors’ predictions, participants showed more difficulties when tested in a virtual than in a real environment. According to the authors, these results suggest that when visual access to the body is absent, as in the immersive virtual reality condition, the boundaries between the self and the environment are blurred; in turn, this blurring could result in more significant sensorimotor conflicts compared to real situations where information about the body is available. These findings may have substantial implications for future research in the spatial cognition and body processing field, where immersive virtual reality is gaining popularity as a tool [35,36].

The studies featured in this Special Issue [9,10,12,13,24,26,31,34] underscore the significance of integrating different perspectives to explore body perception, representation, and the interaction with the surrounding space.

While the articles within the Special Issue offer intriguing research insights, they also raise questions that need further investigation. For example, future empirical research should address if, and to what extent, the interindividual variability in different interoceptive submodalities (e.g., cardiac, respiratory, thermosensory, and affective touch submodalities) could play a role in modulating body representations and the space around the body, considering different developmental stages and different interoceptive dimensions (e.g., accuracy, sensibility, awareness).

We anticipate that this collection will serve as a catalyst for more profound and comprehensive investigations across the healthy lifespan and different clinical conditions that will further advance our knowledge in this intriguing field of research.
